# RCoD: Reputation-Based Context-Aware Data Fusion for Mobile IoT

**DOI:** 10.3390/s25041171

**Published:** 2025-02-14

**Authors:** Samia Tasnim, Niki Pissinou, S. Sitharama Iyengar, Kianoosh G. Boroojeni, Kishwar Ahmed

**Affiliations:** 1 Department of Electrical Engineering and Computer Science, The University of Toledo, Toledo, OH 43606, USA; kishwar.ahmed@utoledo.edu; 2 Knight Foundation School of Computing and Information Sciences, Florida International University, Miami, FL 33199, USA; pissinou@fiu.edu (N.P.); iyengar@cis.fiu.edu (S.S.I.); kgholami@fiu.edu (K.G.B.)

**Keywords:** internet of things, context, data analytics, data fusion, data reliability, reputation, trust, air-quality monitoring, machine learning

## Abstract

The rapid development of mobile sensing technologies (e.g., smart devices embedded with various powerful sensors) has encouraged the proliferation of the Internet of Things (IoT). Although data reliability and accuracy are crucial in many sensor applications (e.g., air-quality monitoring), it is often difficult to ensure these properties. Mobile IoT’s people-centric architecture allows for more inaccurate and corrupted data. In this manuscript, we are addressing the problem of how to predict data more accurately in the presence of malicious participants who inject false data to manipulate the system. Our goal is to recover those missing or imprecise data values from the correlated data streams. To do so, we propose a Reputation-Based Context-Aware Data-Fusion (RCoD) mechanism that is resilient against on–off and data-corruption attacks. Furthermore, the Contextual Hidden Markov Model-based data prediction facilitates more accurate real-time data prediction. We tested the scenarios where most participants were malicious, injecting false data at varied rates. Our method accurately identified the honest participants based on their reported data and context. We empirically evaluate the performance using Beijing’s air-quality dataset. We compared the performance of our RCoD method against four state-of-the-art methods, and the results justify its superiority.

## 1. Introduction

Mobile internet of things (IoT) has been renowned as a state-of-the-art sensing data-gathering epitome [1,2]. The rapid development of mobile sensing technologies (e.g., smart devices embedded with various powerful sensors such as temperature, accelerometer, humidity, and proximity) has encouraged the proliferation of mobile IoT. Mainstream smartphones and recently popular wearable devices such as smartwatches, fitness-tracking devices, and narrative clips are equipped with many sensors. These devices can be used as abundant sources of raw data [3]. In recent years, mobile IoT has gained increased applications in different areas, including transportation, air-quality monitoring, epidemic disease monitoring, reporting from disaster situations, environmental monitoring, and so on [4,5,6,7].

HazeWatch [8], a mobile IoT system, depends on citizen participation for air pollution monitoring. Air pollution has a negative impact on public health. As per the statistics published by the World Health Organization (WHO), 4.2 million premature deaths occur annually due to air pollution. A 9-year-old girl who passed away during an asthma attack is believed to be the first individual to have air pollution recognized as a cause of death [9]. High concentrations of particulate matter with a diameter less than 2.5 μm (PM2.5) in the pollutant air causes cardiovascular or respiratory diseases and cancers. Unfortunately, most people across the world, specifically 91%, inhabit areas where the air pollution levels exceed WHO-defined limits [10]. Agencies such as the National Environment Agency of Singapore are now using HazeWatch every day.

In contrast to traditional sensor networks, where a large number of sensors are required to be deployed to sense data, mobile IoT is open in nature, allowing anyone to participate at any time. In mobile crowdsensing-based IoT applications, the task of sensing is assigned to a person. However, successful information transmission largely depends on multiple factors. Some of these factors are behavioral (lack of time or willingness), and others are due to resource limitations (e.g., network bandwidth and smartphone battery) for performing the sensing task. Also, this people-centric architecture allows both more inaccurate and corrupted data [11]. Malicious participants can easily manipulate the IoT data collection process by reporting fabricated or erroneous data.

Although reliability and accuracy are of utmost importance in many sensor applications (e.g., air-quality monitoring), it is often difficult to ensure these properties in such applications [12]. In the air-quality monitoring application, the sensed pollution data are used to create a pollution map. Imprecise pollution information about an area will mislead people. For instance, due to erroneous pollution data, an asthma patient who prefers a pollution-free route for a walk might be directed to a polluted area. This misdirection will have a negative impact on his/her physical well-being. A malicious user may introduce erroneous air-quality data for a number of reasons. Two such motivations are financial gain and reputation manipulation. Businesses or individuals may falsify air-quality data to save money by avoiding penalties or regulatory inspection. To enhance tourism and business (e.g., restaurants), malicious entities can report poor air quality (by holding the sensor beside a burning cigarette or burning leaves, among others) so that tourists avoid a particular region that belongs to the competitors and become attracted to the attacker’s business area. On the other hand, as an illustration of reputation management, organizations may wish to give the public a more positive impression of the environment by fabricating data to indicate higher air quality. As more cities and organizations use crowdsensing and IoT devices for environmental monitoring, the possibility of such attacks is becoming a greater threat. Thus, trust evaluation is a major issue in these applications to ensure data reliability and integrity. Data reliability refers to the condition when data attains enough completeness to be considered for its goal and context [13,14].

While researchers [1,15,16,17,18,19,20,21] have attempted to improve the quality of the received sensor data, limited research has been done on how sensor context (e.g., sensor model, terrain elevation, wind speed, population density, and user movement during sensing) can be used in sensor selection for data cleaning. In related contemporary work [1,15,22], the authors considered user context for data quality estimation in mobile IoT. However, Gill et al. considered either temporal [16] or spatial relationships [15] among the sensors while developing model-based data-cleaning mechanisms. Because only one type of relationship is considered, these methods did not achieve decent cleaning accuracy and have limited practical impact. The methods failed to exploit the dynamism of the experimental environment while selecting the correlated sensors for data prediction. On the other hand, the authors [1] considered the presence of exactly one mobile user at each point of interest (PoI), which is a limited setting and not practical in real-world scenarios.

Furthermore, the authors did not consider the participants’ malicious behavior. Thus, these works were not able to distinguish the sensing data reported by malicious or careless users. This limitation of the existing works motivates us to design context-aware reputation-based real-time data-fusion algorithms for mobile IoT to ensure data integrity. Our method can detect malicious participants and prevent them from infiltrating the system in real time. Even in the case of high false data injection, our method can ensure data reliability.

In this article, we consider the on–off and data-corruption attack behavior of a malicious participant. A data-corruption attack occurs when a participant sends incorrect data either deliberately or recklessly. The reckless error occurs when a participant heedlessly performs the task of sensing or was caused by a sensor error. In contrast, a malicious participant can intentionally fabricate the sensed data to penetrate the system [20]. On the other hand, an on–off attack means that malicious participants behave good and bad alternatively, hoping that they can remain undetected while hampering data quality [23,24]. In this article, we interchangeably use the terms participant, user, or node to denote a user with sensing capability.

In summary, we introduce the following key contributions: (1) we develop an online method for data quality prediction in mobile IoT that considers the spatiotemporal, contextual, and inter-sensor-category correlations among the participants. The inter-node distance at a specific instance, as well as user context (sensor model, wind speed, and user movement during sensing), are considered in correlated node selection. (2) We are the first to use Contextual Hidden Markov Model (CHMM) for online data prediction in mobility-centric IoT. CHMM has the unique capability of fusing temporal dependence and contextual spatial relationships [25,26,27]. Also, it is a lightweight method and thus suitable for our big-data application. This motivates us to apply CHMM for data prediction in mobile IoT. (3) We consider dynamic spatial granularity while defining the correlated participants for data fusion depending on the application type: spatial stable or geo-sensitive (e.g., caused due to diffusion and dispersion), which was not considered in the earlier works. (4) We present an effective solution, called Reputation-Based Context-Aware Data-Fusion method (RCoD) that is resilient against data-corruption and on–off attackers. We empirically evaluate the performance using Beijing’s air-quality dataset [28]. In this factual dataset, 149 taxis with four types of sensors collect PM2.5, PM10, NO_2_ and humidity data from Beijing for seven days. We compared the performance of our RCoD method against four state-of-the-art works, and the results justify its superiority.

## 2. Related Work

In this section, we discuss the works that are most pertinent to our work. With the recent widespread use of different sensing technologies, we have a great amount of sensor data. Therefore, we must ensure the better quality of data. Recently, authors have placed more importance on data quality of the sensed data. In related contemporary work [1,15,16], the authors considered user context for data quality estimation in mobile IoT. Gill et al. [15,16] designed a context-aware multiple regression-based method for cleaning data streams. They built models of environmental sensor data incorporating context awareness about sampling locations. The historical value of each stream is stored to calculate a run-time standard deviation and to determine outliers. Gill et al. used the three Sigma rule [15]. On the other hand, researchers [16] added context (distance to 3 different locations) to the data stream for prediction and cleaning. Concurrent streams with similar time stamps were processed. The shortcoming of this work is that the authors did not consider any spatial relationship among the data streams.

On the contrary, Peng et al. [29] used unsupervised learning for data quality estimation. This method works after the collection of historical data from all the users; hence, it is not an online method. Trustworthiness has been considered a measure of data quality estimation [11,30,31]. However, Mousa et al. [11] used a synthetic dataset for the experimentation. Moreover, Alswailim et al. [32] proposed a method named Reputation System to Evaluate Participants (RSEP) to cluster participants into three groups based on the sensed data. If the data were within 10% error of the ground truth, they were considered to be correct. The winning group was given a reward in terms of increased reputation, and the reputation value of members belonging to the remaining two groups was reduced. However, the maximum error that RSEP could identify was only 30%. Also, they assumed that ground truth data are known a priori, which is not practical in real-life applications.

CHMM has been used in the area of computer vision [33,34], wavelet domain [27], and image recognition [25]. Target appearance change during tracking is always a challenging problem for visual object tracking [33]. CHMM was also used in dynamic behavior analysis of power distribution networks, equipped with phasor measurement units (PMU), with the aim of providing adequate assistance to diagnostic and control application [26]. Moreover, Bushra et al. [35] incorporated an autoregressive hidden Markov model for filtering out compromised nodes in static wireless sensor networks. Since it is an autoregressive model, it requires high processing capabilities. This method fails to identify malicious nodes when the nodes move.

Chen et al. [36] proposed a deep learning-based model for urban air-quality monitoring. Even though the authors were able to predict and forecast air-quality index values by exploiting spatial as well as temporal models, it required higher processing time and capability. Also, it is completely supervised and cannot work on unlabeled data. On the contrary, a multi-linear regression model was used for forecasting air pollution index [37]. Kumar et al. [38] utilized an autoregressive integrated moving average model for predicting air-pollutant concentrations. Furthermore, Cheng et al. [39] focused on calibration error reduction in air-quality monitoring sensors by utilizing spatial correlation and multi-sensor fusion. A recent research work by Costa et al. focused on utilizing low-cost fixed sensors for air-quality monitoring of Cabo Verde Islands [40]. However, the network structure was static, i.e., the deployed air-quality monitoring sensors did not change their position. Hence, the area coverage was limited, causing data sparsity problems. To overcome the data scarcity of image-based data, Shah et al. designed a synthetic image generator algorithm to imitate factual citizen-based air-quality image data as if it were captured by a cell phone [41]. However, these works neither looked into data reliability issues nor could detect the presence of malicious users in the air-quality monitoring system.

Recently, instead of traditional static wireless sensor networks, the sensing is distributed among a crowd of people, which is termed mobile crowdsensing. This crowdsensing brings heterogeneity in the sensor networks and makes the computation more complex. The most recent work on data quality estimation in mobile crowdsensing was published by Shengzhong et al. [1]. The authors broached real-time data estimation in mobile crowdsensing and proposed a context-aware method for data quality estimation. Due to the involvement of humans, the level of uncertainty in data increases. The limitation of this work is that the authors considered the presence of exactly one mobile user at each point of interest (PoI). Saloni et al. [42] developed LASSO, an infrastructure-less smartphone-based real-time method for monitoring groups of people. In contrast, Kishino et al. [43] mounted sensor nodes on garbage trucks that drive around the city. Their motivation was to detect target events by analyzing vehicle-mounted sensor data streams. The authors used machine-learning methods to achieve so. On the other hand, the author [44] broached a new sampling method named stratified sampling for calculating the mean temperature of a linear area. In this article, only a random waypoint mobility model was considered for the movement of the sensing devices.

Several works focus on the cleaning of data streams. Most of the previous works on sensor data cleaning focused on the reduction of consumed energy. To achieve this reduction, the authors [45,46,47] tried to reduce the inter-node communication. In these works, it was assumed that sensor data are always aggregated during submission. Currently, researchers [1,15,16,18,48] are designing frameworks to deal with big-data services. In the past, the data size was not as big as presently which influences researchers to design and develop scalable mechanisms to correct any inaccuracy in data streams. Liu et al. [48] designed a framework for big-data cleaning. This paper gave direction on how to achieve reliable databases in big-data applications. In order to find similarities between data items, they used context. Moreover, the authors exploited the usage pattern to classify and group data items that are not related contextually. One of the challenging tasks in dealing with big data is to shrink the data size by extracting the irrelevant subset. Dong et al. [49], in contrast, debated that having more data does not always provide more information. During data integration, the proper selection of a reliable source among all available sources results in higher data accuracy.

Another aspect of the research focuses on finding outliers in sensor data streams. There exist different techniques for finding outliers. In order to find global outliers in the data, Branch et al. [47] proposed a distance-based ranking method. In contrast, Tasnim et al. [50] proposed a semantic-aware method for trajectory outlier detection. The other existing methods for finding outliers in sensor data are geometry-based [51], polygon-based spatial outlier detection [52], clustering-based [53], kernel density-based [54] and histogram approach [55]. Jeffrey et al. [56] and Sharma et al. [57] proposed a mean-based method for data prediction in sensor data streams. In contrast, Vzliobaite et al. [58] developed a fault-tolerant model based on linear regression.

On the other hand, Bosman et al. [59] tried to answer the question if adding more neighbors makes the anomaly detection perform better. This paper considered static sensor nodes and the authors varied the neighborhood size by changing the communication range of the sensors. Also, Zhang et al. [60] proposed a data-cleaning method for environmental sensing, which was based on incrementally adjusted reliability of individual sensors. With the advance of time, they incrementally adjusted the reliability of each sensor depending on the sensing data accuracy. Furthermore, Huang et al. [19] showed that using a reputation framework helped to remove non-colluding malicious attackers. However, the authors assumed that data are coming from every spatiotemporal unit, which is not practical in real-world scenarios. In contrast, a correlated sensor-based data-fusion mechanism is presented in [20]. The method cannot detect the on–off attack. If the malicious user behaved alternatively as honest and malicious, the method fails to identify it, and thus, the data prediction accuracy deteriorates acutely. Furthermore, the authors assumed that the majority of the participants were honest. The CDR method [20] could tolerate up to 30% of malicious participants. Since only equal-sized grid-based spatial discretization was used in this paper, it failed to exploit the dynamism of the experimental environment.

In order to lower trust computing overhead, communication overhead, and communication delay in the trust-evaluation process, Liang et al. presented a dependable trust computing method based on multi-source feedback and data fusion in fog computing [61]. On the other hand, to address risks in the data-gathering process and guarantee data quality, Lv et al. suggested a wireless sensor network-based data security collection trust-evaluation method [62]. With the objective of assessing the credibility of users during the data-fusion process in the context of big data, Wang et al. created an efficient trust-evaluation technique [63]. An effective and feasible data-fusion trust system for multi-source and multi-format data interchange in heterogeneous networks was proposed by Qiu et al. after they designed data-fusion trust architectures with varying trust levels [64]. In addition, Yu et al. fully utilized the universally applicable features of the data-fusion process, assessed the ordinary group’s dependability by counting the number of communications between the ordinary group and the absolute trust group, and measured various group attribute similarities [65].

Gao et al. presented a multi-criteria trust-evaluation system for information sources [66]. This mechanism incorporated feedback-based trust factors, relationship-based trust factors, behavior-based trust factors, and identity-based trust factors. Moreover, Braggato et al. demonstrated the effect of false data injection on high renewable energy source power systems. They used the false data-injection rate between 25% and 75% [67]. Chen et al. [12] designed a 3-tier hierarchical trust management protocol called IoT-HiTrust, proposing the metrics of honesty, cooperativeness, and community-interest as social trust metrics. IoT-HiTrust could handle attacks such as bad-mouthing, ballot-stuffing, and self-promoting attacks for air pollution detection and augmented map travel assistance. In contrast, the authors aimed to make sure that the data provided by a mobile device did, in fact, match the data that the device’s sensors actually reported [68,69,70]. They assume that a malicious user or program may alter phone software (tested on Nokia N800 devices with SecFlecks) and tamper with sensor data. An auxiliary trusted platform module (TPM), which attests to the integrity of sensing devices, is the core of their solutions. However, the solutions for TPM-enabled cell phones are not yet widely deployed since they have not yet been mass-manufactured. Furthermore, when a user physically causes interference that alters sensor readings, the TPM is unable to detect such an attack.

In contrast to all these existing related contemporary works that are poorly resilient against a large number of malicious participants, we intend to design a reputation-based real-time data-fusion mechanism for mobile IoT data streams that can exploit the dynamism of the experimental environment using Contextual Hidden Markov Model yielding a lower data prediction error rate.

## 3. Problem Formulation

There is data imprecision or missing values in the crowdsensing applications due to frequent loss of communication, hardware error or malicious intention of the carrier. Thus, it is important to detect those data imprecision and predict those incorrect and/or missing values. The problem being addressed in this article is predicting data more accurately in the presence of malicious participants who inject false data to vandalize the system. Our goal is to recover those missing or imprecise data values from the correlated data streams.

Let us assume that there are *N* participants identified as trusted from the reputation system. Thus, the whole data matrix has the size N×T, where *T* is the duration. Matrix $V^{\left(N\times T\right)}$ represents all time series (*T*) values from the *N* trusted participating sensors. Matrix *E* keeps track of the missing data. If there is a missing value or erroneous reading ($V_{i,j}$) from $i^{th}$ participant during a particular timestamp *j*, $E_{i,j}=1$, otherwise $E_{i,j}=0$. The size of the error matrix is N×T, the same as *V*. The problem of missing value prediction (M) is defined as follows.

Given M={V,E,C}, estimate ${\tilde{V}}_{ij},\;\mathrm{for}\left(i,j\right)\in \left(i,j\right):E_{i,j}=1$. Where $V\in R^{N\times T}$ represents the *T* timeseries data from *N* crowdsensing participants, $E\in R^{N\times T}$ represents the error matrix and $C\in R^{N\times N}$ is the contextual matrix. The matrix *C* denotes the pairwise contextual correlation among the participants. The data range of matrix *C* is [−1,1]. Here, 0 denotes no correlation, and a higher value insinuates a higher correlation. Hence, it has a size of N×N. Our hypothesis is that a context-aware reputation-based data-fusion mechanism will facilitate the accurate detection of malicious participants exhibiting on–off and/or data-corruption attacks and eventually ensure more accurate data prediction in terms of less data prediction error.

## 4. Reputation System

In this section, we describe our reputation and trust distribution mechanisms. We discuss the attack model. Moreover, different components of the trust computation module are discussed in detail.

### 4.1. Malicious Entities and Attacker Strategies

No encryption mechanism is applied in the mobile crowdsensing-based IoT application during the data collection and transmission phases. Anyone can participate in the sensor data collection procedure, making it lightweight and more scalable at a lower cost. However, a malicious participant can disrupt the system by launching the on–off attack and/or data-corruption attack. Due to the absence of an authentication mechanism, a malicious participant can inject false data easily.

#### 4.1.1. On–Off Attack

It is a sophisticated attack and harder to detect and prevent. An on–off attack means that malicious participants behave good and bad alternatively, hoping that they can remain undetected while hampering data quality [23,24]. Most of the state-of-the-art methods fail to detect the on–off attack and thus cannot ensure data accuracy in the presence of on–off attackers. In this type of attack scenario, a participant is aware of honest behavior. In other words, s/he knows the original sensing data of a particular spatiotemporal unit. They report the correct information for a long time to attain higher reputation value. Then, these malicious participants inject false data similar to high spike to manipulate the sensor data. Their motivation is to change the aggregated data and as a result, result in incorrect decisions.

#### 4.1.2. Data-Corruption Attack

A data-corruption attack occurs when a participant sends incorrect data either deliberately or recklessly. The reckless error occurs when a participant heedlessly performs the task of sensing or is caused by a sensor error. In contrast, a malicious participant can intentionally fabricate the sensed data to penetrate the system [20]. For example, in air-quality monitoring, a malicious participant may hold the sensor beside a burning cigarette or place it over sand instead of facing the air. Thus, the reported data will not represent the actual air quality. We considered two types of false data-injection rates. First, the malicious entities inject false data at a constant rate throughout the experimental duration. In the second, the rate of false data injection by a malicious user varies at different time instances. However, in a data-corruption attack, if a participant is malicious, s/he does not behave as an honest participant at any time. This is the major difference between an on–off and a data-corruption attack. The false data-injection rate is randomly selected from the range of 30% to 75%.

We assume the devices are properly calibrated before the experimentation, i.e., participating in the sensing task. Thus, calibration error is out of the scope of this article. We focus on the data inaccuracy caused by participants inadvertently or intentionally.

### 4.2. RCoD Mechanism

**Figure 1:**
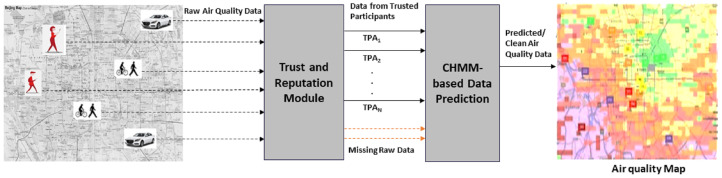
Overall Architecture of RCoD.

Figure 1 illustrates the main components of our RCoD mechanism. Sensed air-quality data are reported by various participants to the server. After that, all these contributions are input to the Trust and Reputation module. Here, the contributions’ trustworthiness is analyzed considering different properties (Section 4.3, Section 4.4 and Section 4.5). Each participant’s reputation is calculated, which reflects historical behavior (Section 4.6). Based on their reputation, trusted participants are identified. Next, data from trusted participants (with higher reputation scores) identified in the previous step (Section 4.6) are input to the Contextual Hidden Markov Model. Also, the data streams containing missing data are taken as input. Finally, we describe the CHMM-based data prediction methodology (Section 5). These accurate predicted data are used to generate pollution maps as depicted in Figure 1. Green denotes healthy air quality, yellow for moderate, and red denotes unhealthy regions on the pollution map.

### 4.3. Dynamic Set of Trusted Participants

In most of the state-of-the-art methods, the ground truth value is calculated from the data reported by all the participants. In contrast, we dynamically update the set of trusted participants. The ground truth value is calculated from the data reported by the trusted participants. Since we periodically update this set of trusted participants, it is ensured that the trusted set does not include malicious participants who are trying to forge data. Thus, the data accuracy and robustness of the system is maintained. Our mechanism can detect malicious users and mitigate the false data injected by these users.

In the initialization phase, when there is no historical data, the similarity between contributions received from multiple participants is calculated. If $C_{i}^{k}$ is the sensor data of type *k* provided by participant *i*, then its similarity with all other data of type k contributed by the other participants regarding the spatiotemporal unit is calculated. The normalized average difference is calculated to be used in the exponential-based initial contribution score generation. The contribution scores range between 0.36 (=$e^{-1}$) and 1. This score is an input to the reputation table. Then, these initial contribution scores are sorted in descending order. The top TPA participants are selected from the sorted list to be declared as the set of trusted participants. The number TPA is application-dependent. We calculate the ground truth value using Equation (1) from the data reported by participants belonging to the trustedSet.

(1) $$Groundtruth=\frac{\sum C_{i}}{\left|trustedSet\right|},\;\forall i\in trustedSet$$

Here, i denotes the participant ID, $C_{i}$ is the contribution data provided by participant i.

Now, we briefly describe Algorithm 1: the formation of the initial trusted set. It takes the data contributions made by all the participants on the initial duration (epoch = [1, initEpoch_end]) to return an initial set of trusted participants. The value of initEpoch_end is application-dependent. First, all the participants who contributed data on the initial duration are listed. Then, the difference between the data reported by different participants at the same epoch (temporal unit) is calculated (lines 6–9). In line 10, the average difference value is calculated for a specific participant i and stored in the array diff (line 10). Next, in line 14, the normalized difference is calculated. For this purpose, the minimum and maximum values of the array diff are identified. Moreover, the reputation of each participant is calculated and stored in the global reputation table. This table contains three columns. The first column contains the participant ID, the second contains the initial_contribution score, and the final column is dedicated to storing the reputation score. The details of reputation score calculation procedure is described in Section 4.6. In lines 13–16, the reputation table is updated with the calculated information regarding day 1 participants.   
**Algorithm 1:** Formation of Initial Trusted Set **Input:** Participant Contributions, initEpoch_end    1:**Initial Trusted Set**    2:**for** epoch=1:initEpoch_end **do**    3:    participantSet←id    4:    participantLen=|participantSet|    5:    **for** i=1:participantLen **do**    6:        **for** j=1:participantLen **do**    7:            $C_{ij}=abs\left(data_{i}-data_{j}\right)$    8:            $Total\_C_{ij}=Total\_C_{ij}+C_{ij}$    9:        **end for**  10:        $diff\left(i\right)=\frac{Total\_C_{ij}}{participantLen}$  11:    **end for**  12:    **for** p=1:participantLen **do**  13:        reputation_table(p,1)←participantSet[p]  14:        $Norm\left(p\right)\leftarrow \frac{diff(p)–min(diff)}{max(diff)-min(diff)}$  15:        $reputation\_table\left(p,2\right)\leftarrow e^{-Norm(p)}$  16:        reputation_table(p,3)←        Algorithm 3(p,reputation_table)  17:    **end for**  18:    **Select Trusted Participants**  19:    $trusted_{no}=\frac{participantLen}{2}$  20:    sortedList=sort(reputaion_table,descend)  21:    trustedSet←top(reputaion_table,trusted_no)  22:**end for**

Next, in the Selected Trusted Participant method, the trusted set is defined. First, the size of the set is defined as half of the total number of participants (line 19). Then, the participants are sorted in descending order based on their reputation value. Finally, the top predefined number of participants is selected and assigned to the trusted set. We compiled different notations and their description in Table 1.

### 4.4. Trust Value Assignment

**Figure 2:**
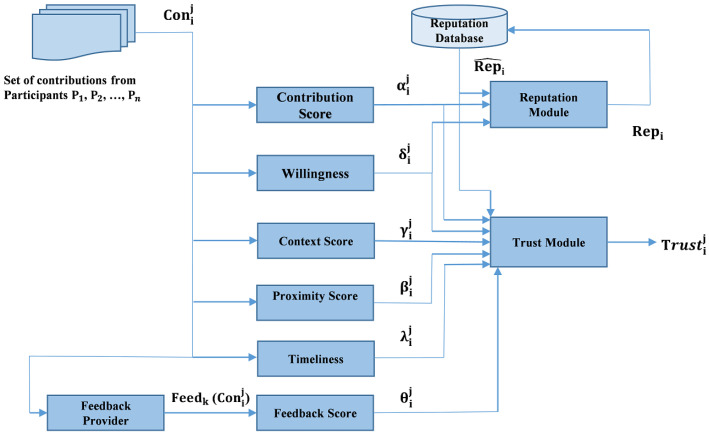
System Model.

Figure 2 depicts the overall system that includes the trust and reputation module.

#### 4.4.1. Contribution Score

In this section, a score is assigned for the recent data contribution made by a participant. pc denotes the participant count, the number of participants who contributed data. The data are compared with the reported data about the same spatiotemporal unit from trusted participants. The sensor data of type *j* contributed by a participant *i* is compared against the reports of the same type from the trusted set of participants. The difference values are normalized using Equation (4). Here, $dif_{i}^{j}$ is the absolute difference for participant i with sensor data type j. $min(dif_{i}^{j})$ and $max(dif_{i}^{j})$ denote the minimum and maximum difference among all the participants during that epoch. After normalization, the difference values belong to the range [0,1]. The value 0 means the contribution is the same as the trusted participants. The normalized score is input to the exponential equation Equation (5) to calculate the contribution score ($\alpha_{i}^{j}$).

The output value of Equation (5) has the maximum value of 1 and minimum value of $e^{-1}$.

(2) $$data_{trustedSet}=\frac{\sum_{k=1}^{\left|trustedSet\right|}data_{k}}{\left|trustedSet\right|}$$

(3) $$dif_{i}^{j}=abs\left(data_{i}^{j}-data_{trustedSet}\right),\forall i\in \left\{1,2,\cdots ,pc\right\}$$

(4) $$Normdif_{i}^{j}=\frac{dif_{i}^{j}-min\left(dif_{i}^{j}\right)}{max\left(dif_{i}^{j}\right)-min\left(dif_{i}^{j}\right)}$$

(5) $$\alpha_{i}^{j}=e^{-Normdif_{i}^{j}}$$

#### 4.4.2. Proximity Score

If the sensing data type does not fluctuate much based on distance and is stable throughout a wide spatial area (e.g., grid), then the proximity score is of Boolean type with a value of either 0 or 1. If two of the participants’ (e.g., $P_{i}$ and $P_{j}$) locations belong to the same grid, then they will have similar sensed values. Here, i and j have the value from 1 to the total number of participants. The proximity score is calculated using Equation (6).

(6) $$\beta_{i}=\{\begin{array}{cc} 1, & grid\left(P_{i}\right)\cap grid\left(P_{j}\right)\neq \phi ,\\ 0, & \mathrm{otherwise}. \end{array}$$

On the other hand, some of the applications are location-sensitive. The value changes significantly with the increase in the distance between the source and the participant who reports the sensed value. In reality, the nature of the applications, such as diffusion and dispersion, play a significant role in the data variation. For example, in pollution detection or noise monitoring applications, a participant located close to the data source will be able to render the most accurate data of the phenomenon. For these highly location-sensitive sensing applications, we calculate the inverse of the Gompertz equation for assigning proximity score to each data contribution (Equation (9)). The L2-norm, calculated using Equation (7), is input to Equation (8).

(7) $${\left||L|\right|}_{2}=\sqrt{{\left(target_{x}-x_{i}\right)}^{2}+{\left(target_{y}-y_{i}\right)}^{2}}$$

(8) $$exponent_{i}=relevance_{b}\times e^{-(relevance_{c}\times ||L|{|}_{2})}$$

(9) $$\beta_{i}=1-relevance_{a}\times e^{-exponent_{i}}$$

There are three parameters for the inverse Gompertz function $relevance_{a}$, $relevance_{b}$, and $relevance_{c}$. The parameter $relevance_{a}$ controls the higher asymptote on the *y*-axis. The displacement on the *x*-axis is controlled by the parameter $relevance_{b}$. The final parameter $relevance_{c}$ controls the function’s decay rate. ($target_{x},target_{y}$) is the target sensing location and ($x_{i}$, $y_{i}$) denotes a current location of the participant.

#### 4.4.3. Rating Score Validation

Periodically, feedback from other participants is gathered for the verification of contributions/sensed data provided by a participant about a particular geographic location. While executing the verification task, the users with a higher reputation score than the target participant are selected. If the data variance is within a tolerable range, then the system assures that the target participant is trustworthy in that particular time instance. In contrast, if most of the higher reputable participants report that the data contributed by the target participant does not match with the actual sensed value of that spatiotemporal unit, a negative feedback score is assigned to the target participant. Since the aggregated feedback (Equation (10)) is considered for assigning the final feedback score, it is resilient against unfair rating attacks. An individual cannot successfully disrupt the system’s trustworthiness by providing negative feedback to an honest participant. Consequently, the on–off attack of malicious participants is prevented.

(10) $$\begin{array}{c} \theta_{i}=\frac{\sum_{f=1}^{Num_{FP}}Rep\left(P_{f}\right)\times Feed_{f}\left(P_{i}\right)}{\sum_{f=1}^{Num_{FP}}Rep\left(P_{f}\right)},\\ \forall f\in Num_{FP}:Rep\left(P_{f}\right)\geq Rep\left(P_{i}\right) \end{array}$$

Here, $Num_{FP}$ is the number of feedback provided, and $P_{i}$ denotes the participant for whom the feedback is collected. In Equation (10), the reputation value of the feedback provider is used as a weight in the feedback score (θ) calculation. Feedback from a higher reputable participant has a higher influence on the calculation of the combined feedback score.

#### 4.4.4. Willingness

A number of non-missing data provided by the participant are among all the contributions during a certain duration. In the dataset, if a participant is in a spatiotemporal unit but does not report data for consecutive time instances, it means the participant lacks the willingness to participate. We assume for this work that battery level is not a reason for data inconsistency or missing data. The smartphones used for the sensing purpose had enough energy storage during the experiment.

(11) $$\delta_{i}^{j}=\frac{\sum_{t=1}^{cur_{epoch}}\left|C_{i}^{t}\setminus empty\left(C_{i}^{t}\right)\right|}{|C_{i}^{t}|}$$

In Equation (11), the ratio of the number of non-empty contributions and total contributions made by participant i of data type j during the previous t epochs is calculated. t has the value from 1 to the current epoch.

#### 4.4.5. Context Score

We compare the similarity of the contextual value of a participant with the context value of the trusted participants who reported a similar type of sensor data at the same time instance. The context value of sensor data type *j* contributed by a participant *i* is compared against the context value of the trusted set of participants. The difference values are normalized using Equation (14). After normalization, the difference values ($Contextdif_{i}^{j}$) belong to the range [0,1]. The value 0 denotes that the context value of the participant is the same as the trusted participants. Then, the normalized score ($ContextNormdif_{i}^{j}$) is input to the exponential equation Equation (15) to calculate the context score.

(12) $$Context_{trustedSet}=\frac{\sum_{c=1}^{\left|trustedSet\right|}Context_{c}}{\left|trustedSet\right|}$$

(13) $$\begin{array}{c} Contextdif_{i}^{j}=abs\left(Context_{i}^{j}-Context_{trustedSet}\right),\\ \forall i\in \{1,2,\cdots ,pc\} \end{array}$$

(14) $$\begin{array}{c} ContextNormdif_{i}^{j}=\\ \frac{Contextdif_{i}^{j}-min\left(Contextdif_{i}^{j}\right)}{max\left(Contextdif_{i}^{j}\right)-min\left(Contextdif_{i}^{j}\right)} \end{array}$$

(15) $$\gamma_{i}^{j}=e^{-ContextNormdif_{i}^{j}}$$

where $\gamma_{i}^{j}$ denotes the context score of participant i with sensor type j. pc is the count of the participants co-located in the same spatiotemporal unit.

#### 4.4.6. Timeliness

This property checks if the participant reported data in a timely manner. The difference between the task assigned ($t_{a}$) and the data reported ($t_{r}$) is taken into consideration. If the difference is greater than the application-dependent threshold $t_{duration}$, then those data are stale, and the timeliness score for the participant will be zero. On the other hand, if the difference is low, it insinuates that the participant carried out the sensing task expeditiously. We calculate the timeliness score (λ) using the inverse Gompertz function. If $t_{a}$ is the task assignment time and $t_{r}$ is the time when the data were reported, then

(16) $$t_{diff}=t_{r}-t_{a}$$

(17) $$\lambda_{i}=\{\begin{array}{cc} a\times e^{-be^{c\times t_{diff}}}, & t_{diff}\leq t_{duration},\\ 0, & \mathrm{otherwise}. \end{array}$$

**Figure 3:**
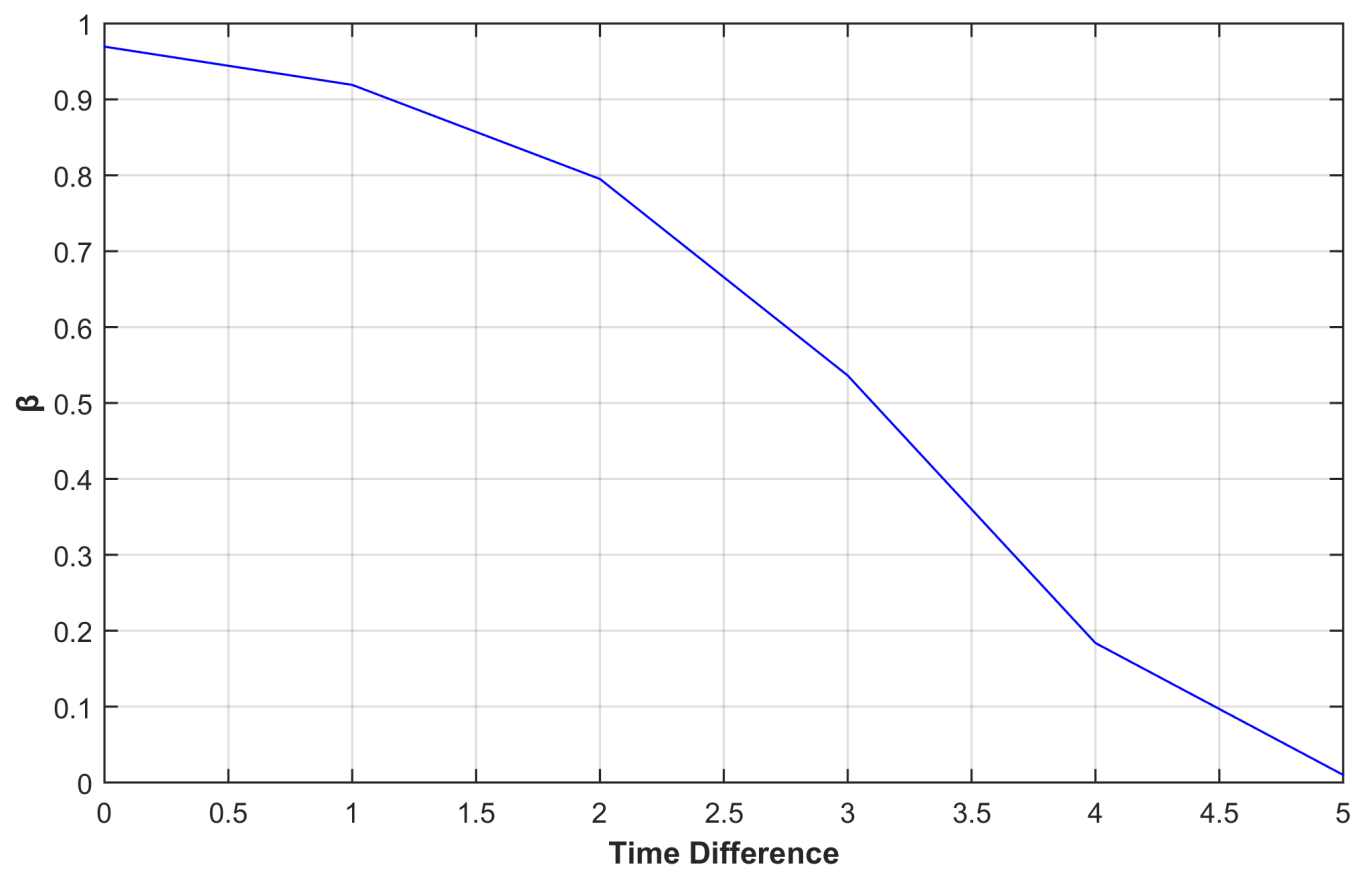
Timeliness Score (λ) vs. Time difference using Inverse Gompertz Function.

There are three parameters for the inverse Gompertz function (Equation (17)): ’a’, ‘b’, and ‘c’. The parameter ‘a’ controls the higher asymptote on the *y*-axis. The displacement on the *x*-axis is controlled by the parameter ‘b’. The final parameter ’c’ controls the function’s decay rate. When the value of $t_{diff}$ is equal to $t_{duration}$, the timeliness score is almost equal to zero, such as 0.01 as visible in Figure 3.

### 4.5. Trust Level Mapping

The trust level of each contribution made by a participant is calculated. It is a combined metric consisting of the above-mentioned six property values: contribution score, proximity score, rating score, context score, willingness, and timeliness. The reputation value (rep) of the previous epoch is also included in the combined trust level calculation as shown in Equation (18).

(18) $$\begin{array}{c} Trust\left(C_{i}^{j}\right)=w_{1}\times \alpha_{i}^{j}+w_{2}\times \beta_{i}+w_{3}\times \theta_{i}+\\ w_{4}\times \gamma_{i}^{j}+w_{5}\times \delta_{i}^{j}+w_{6}\times \lambda_{i}+w_{7}\times rep \end{array}$$

where $\sum_{i=1}^{7}w_{i}=1$. We assign an initial trust value of 0 to all participants. As a result, a new participant cannot simply inject false data.

The heterogeneous trust distribution method is summarized in Algorithm 2. It takes data contributions made by participants, starting epochs, and ending epochs as inputs. It is an iterative process that calculates the trust score for all the participants who reported data in that epoch. The iteration continues from the input value epoch_begin and finishes at the epoch_end. First, all the participants who reported data in the considerable epoch are included in a list. Then, for each participant belonging to the list, the contribution score is calculated using Equation (5) (line 5). In the next step, based on the application type (e.g., location-sensitive or location-stable), the relevance score (β) is calculated. For a geo-stable application, the equation used is Equation (6), otherwise Equation (8) is used (lines 5–9) for β calculation. The rating score (θ), context score (γ) and timeliness score (λ) are calculated calling Equations (10), (15) and (17), respectively (lines 11–13). Next, the reputation table is consulted to obtain the reputation score of the participant. The value assigned to the rep is the old reputation value of the participant. To update the reputation table for the current contribution, Algorithm 3 is called in line 15. Finally, the trust value of the contribution made by a participant of type j is calculated in line 16. The total aggregated value of $w_{1},w_{2},w_{3},w_{4},w_{5},w_{6}$ and $w_{7}$ is equal to 1.
**Algorithm 2:** Heterogeneous Trust distribution System **Input:** Participant Contributions, epoch_begin, epoch_end    1:**for** epoch=epoch_begin:epoch_end **do**    2:    $P_{List}\leftarrow ID$    3:    **for all** $P_{i}\in P_{List}$ **do**    4:        $\alpha_{i}^{j}\leftarrow$ Equation (5)    5:        $reputation\_table\left[P_{i},2\right]\leftarrow \alpha_{i}^{j}$    6:        **if** geo-stable application **then**    7:            $\beta_{i}\leftarrow$ Equation (6)    8:        **else**    9:            $\beta_{i}\leftarrow$ Equation (8)  10:        **end if**  11:        $\theta_{i}\leftarrow$ Equation (10)  12:        $\gamma_{i}^{j}\leftarrow$ Equation (15)  13:        λ← Equation (17)  14:        $rep\leftarrow reputaion\_table[P_{i},3]$  15:        $reputation\_table[P_{i},3]\leftarrow$ Algorithm 3$\left(P_{i},reputation\_table\right)$  16:        $Trust\left(C_{i}^{j}\right)=w_{1}\times \alpha_{i}^{j}+w_{2}\times \beta_{i}+w_{3}\times \theta_{i}+w_{4}\times \gamma_{i}^{j}+w_{5}\times \delta_{i}^{j}+w_{6}\times \lambda_{i}$        $+w_{7}\times rep$  17:    **end for**  18:**end for**

### 4.6. Reputation Score

The reputation score is dependent on the contribution score and willingness of a participant. It insinuates the historical behavior of a crowdsensing participant. In the reputation score calculation procedure, a higher punishing score for incorrect contribution than a reward score ensures the degradation of the reputation score of a malicious participant trying to vandalize the system performance through exploiting an on–off attack. Even if s/he gained a high reputation due to showing honest behavior through providing correct sensor data, because of recent incorrect contributions, her/his trust score, as well as the reputation score, will fall below the threshold value. Hence, the participant will not be included in the trusted list for future time instances. When the reputation score falls below the threshold, our system ensures that the participant provides a longer period of correct contributions to regain the honest status.

Now we briefly describe Algorithm 3: Reputation Computation. It takes as an input participant ID the current reputation table and returns the updated reputation value for that participant. The reputation value for each participant is initialized at 0. First, iteratively, the participant ID is searched for in the input reputation table (lines 1–3). The old reputation value, which is obtained from the third column of the reputation table, corresponding to that participant, is recorded in the old_reputation variable. Furthermore, the contribution value is accessed from the second column of the reputation table and compared with the predefined threshold value (lines 5–6). If the value is greater than the threshold, it insinuates the correct contribution made by the participant. Thus, a reward value is added to the old reputation value. However, the reputation value cannot exceed the highest value of 1.0. To ensure this, the minimum of new calculated reputation plus 1 is selected as the new_reputation in lines 6–7. In contrast, if the contribution score is below the threshold, it insinuates that the participant reported incorrect data. Thus, a punishment score is applied over the past reputation value. Again, to maintain the minimum reputation score of 0, a maximum of 0 plus calculated reputation is selected and assigned as new_reputation (lines 8–9). Otherwise, due to multiple punishments, a reputation value achieves a negative value. Finally, the reputation table is updated with the new_reputation value.
**Algorithm 3:** Reputation computation **Input:** Id, reputation_table **Output:** Reputation value (rep)    1:dataLen←|reputation_table|    2:**for** l=1:dataLen **do**    3:    **if** reputation_table[l,1]==Id **then**    4:        $old_{r}eputation\leftarrow reputation\_table\left[l,3\right]$    5:        contribution←reputation_table[l,2]    6:        **if** $contribution\geq threshold\wedge willingness\geq w_{threshold}$ **then**    7:            new_reputation=min(1,(old_reputation+rewards_val))    8:        **else**    9:            new_reputaion = max(0,(old_reputation−punish_val))  10:        **end if**  11:        Reputation_table[l,3]=new_reputation  12:    **end if**  13:**end for**  14:returnrep

## 5. Reputation-Aware Data Prediction Methodology

In this section, the reputation-aware data prediction methodology is discussed. Data from trusted participants identified by the reputation module, discussed in the earlier section, are consulted in the Contextual Hidden Markov Model-based missing data prediction methodology.

### Contextual Hidden Markov Model

**Figure 4:**
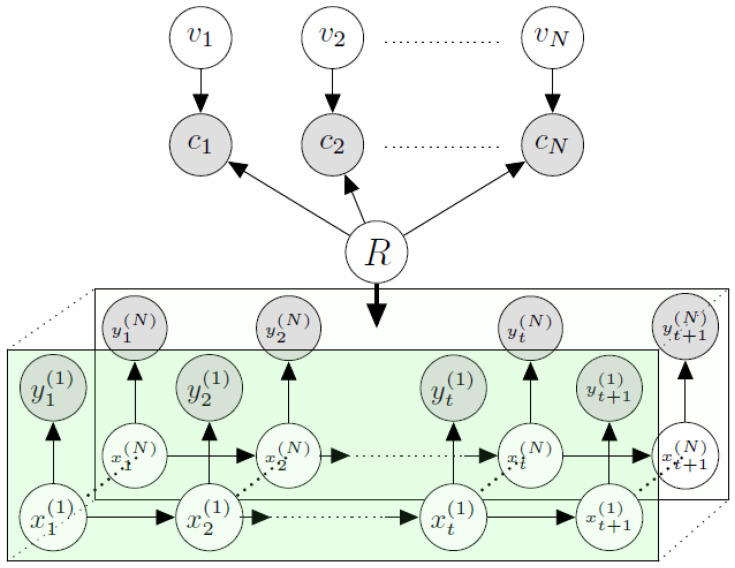
Contextual Hidden Markov Model graph diagram.

In the traditional hidden Markov model (HMM), there is only a temporal relationship; by adding the contextual layer represented by $c_{1},c_{2},\ldots ,c_{N}$, we incorporated spatiotemporal and contextual dependence. According to the definition of HMM, there exists a hidden Markov process $x_{t}$, and an observation $y_{t}$ is controlled by $x_{t}$[71]. Here, $s_{1},s_{2},\ldots ,s_{N}$ denotes different participants who reported data. In Figure 4, the lower diagram enclosed by the rectangle is similar to traditional HMM. The observation value ($y_{t}$) is dependent on $x_{t}$. Here, $x_{t}$ denotes the hidden state at epoch *t*. Epoch is the temporal unit. In Figure 4, each row in the lower enclosed box is dedicated to an individual participant who reported data. For example, the second row from the bottom is dedicated to all the data samples reported by participant ID = 2 at different time instances (epoch = 1,2,…,T). Here, the final epoch is denoted by *T*. The reported data matrix *Y* has the dimension of N×T. *N* is the number of participants who reported data during the time instances commencing at 1 and ending at *T*. On the other hand, the size of the context matrix *C* is N×N.

We incorporate a new hidden observation dependence vector OV and hidden contextual dependence vector CV. For all continuous data streams, the general Markovian dependence ($y_{t}|x_{t}$) is replaced by ($y_{t}|x_{t},OV$). Here, the observed correlation has a combined influence of OV and CV, and thus, the context matrix *C* can be represented using the following conditional probability p(C|CV,OV). The complete likelihood of our proposed CHMM can be noted as,

(19) $$\prod_{t=2}^{T}p\left(x_{t}|x_{t-1}\right)\prod_{t=1}^{T}p\left(y_{t}|x_{t},OV\right)\prod_{j=1}^{N}p\left(C_{j}|CV_{j},OV\right)p\left(OV_{j}\right)$$

In Equation (19), the first product depicts the temporal dependence, the second represents observation, and the final one is for context.

We calculate the missing data using Algorithm 4. It takes the epoch, participant ID, and TrustedSet of participants as input. At first, it is checked if there is any missing data. If the data stream is complete, no further action is required (lines 2–3). Otherwise, the missing data are predicted using the CHMM model. The CHMM is applied to the set of trusted participants and the node that contains missing data. The returned predicted value is assigned to the variable $cm_{0}$ in line 6. In most cases, CHMM is able to return the predicted value. If $cm_{0}$ is empty, it means there is no correlated sensor that contains data. In that case, temporal interpolation is applied to predict the missing data. Finally, the missing data of the input participant are replaced with the predicted value of $cm_{0}$.
**Algorithm 4:** Missing Value Estimation **Input:** time, participant_id, TrustedSet    1:t∈time&s∈participant_id    2:**if** data $sen_{t}^{\left(s\right)}$ is available **then**    3:    continue    4:**else**    5:    $\forall s^{trusted}\in TrustedSet_{t}$    6:    $cm_{0}\leftarrow CHMM\left(sen_{t}^{\left(s^{trusted}\right)},sen_{t}^{\left(s\right)}\right)$    7:    **if** $cm_{0}=empty$ **then**    8:        Predicted_data = Temporal Interpolation    9:    **else**  10:        $\mathrm{Estimated}\;\mathrm{value}\;\mathrm{of}\;sen_{t}^{\left(s\right)}=cm_{0}$  11:    **end if**  12:**end if**

## 6. Performance Evaluation

In this section, we describe our experimental setup and environment. We also describe the dataset we used and the different performance measures that we considered to evaluate the accuracy of our proposed method. We have used Beijing’s air-quality data [28], published by Microsoft Research, to implement our algorithm.

### 6.1. Dataset Description

**Figure 5:**
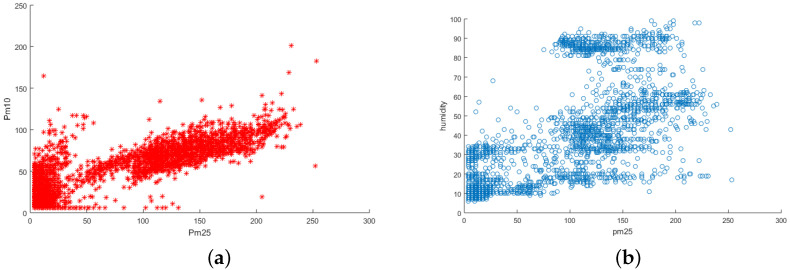
(**a**) Correlation of PM2.5 and PM10. (**b**) Correlation of PM2.5 and humidity.

A total of 149 taxis with four types of sensors collect particulate matter with a diameter under 2.5μm (PM2.5), PM10, NO_2_, and humidity data from Beijing for seven days. The air-quality data were collected from an area of roughly 120 km by 150 km. The duration was seven days (149 h). We assume that the participants are aware of the area where the sensing will take place. Also, the correlation between the different sensors is known. For our experiment, we considered PM2.5 as the target sensor data type and three types of real correlated sensor data (PM10, NO_2_, and humidity). In Figure 5a, the correlation between PM2.5 and PM10 is shown. It can be observed that PM2.5 and PM10 displays linear correlation. In contrast, PM2.5 and humidity are non-linearly correlated as depicted in the Figure 5b.

### 6.2. Simulation Setting

In the original dataset, all the participants are honest. Thus, in order to introduce impurity in the sensed data, we incorporate continuous or random errors in the original data. We have considered two types of data impurity: in the first method, the data error percentage for a taxi is unchanged throughout the time period. In the second set of experiments, we consider a random error rate for a single node. We used MATLAB and Python programming languages for the implementation of our algorithms.

#### Assumptions for False Data Injection

We considered data-corruption and on–off attacks. Similar to the related contemporary works that do not consider colluding among the participants, we had to assume that malicious users inject false data individually. Unlike most of the state-of-the-art methods, the requirement of the presence of a fixed trusted participant for providing ground truth has been relaxed in our method because the presence of a trusted participant at all time instances is not realistic.

We synthetically injected false data in the original data streams to imitate the false data-injection attack. We did two different experiments. At first, the false data-injection rate for an individual participant remained unchanged throughout the experiment. In contrast, the error rate has been varied at different time instances for a participant in the other test cases. For the on–off attack, the false data injection was performed after a long duration of time so that the participant could gain a high reputation value to be considered to be a trusted participant. After that, the original data are intermittently replaced with false data to imitate malicious behavior.

We assume the malicious participants do not collude among themselves to infiltrate the data collection procedure. Also, it is assumed that the participants do not perform trial-and-error attacks, which is a sophisticated attack. In that type of attack, malicious participants can learn different reputation parameters used in the data trustworthiness analysis. Like most of the related contemporary works, we assumed that the malicious participants do not have the chance or, in other words, do not have enough time to guess the system parameters in order to fool the system. Our method makes data-injection attacks harder, but it is breakable by collusion among the malicious participants. In our future work, we will consider collusion attacks.

We tested the performance of our algorithm for different levels of erroneous data from malicious users. We also varied the knowledge level of the participants in regard to the experimental environment to imitate sophisticated data manipulation by a malicious crowdsensing participant. In the first test, we assumed the participants did not have any prior knowledge about the experimental environment. The data error ranged from 25% to 75%. One group of malicious participants reported a fixed percentage of error throughout the experiment. This type of error occurs when there is any technical issue in the sensors or the sensor is placed in a covered area during the execution period. In the second experiment, we consider that the malicious participant has extended knowledge about the sensing area. Thus, these participants try to change the sensing data by adding noise to the air-quality data of that particular spatiotemporal unit. Moreover, to imitate the on–off attack, we applied random data error ranging from 25% to 75% on a participant’s reported data stream after a long display of honest behavior. To bring randomness to the behavior, there was no data error in some of the epochs (temporal units). Table 2 shows different parameter values that we used in the experiment.

### 6.3. Results and Analysis

We calculated Mean Absolute Error, Accuracy, Precision, Recall, F1 Score, AUC, Specificity, and Root Mean Square Error for the performance evaluation. We tested with the presence of different percentages of malicious participants. Most of the state-of-the-art methods assume the presence of malicious participants ranging from 20% to 40%. However, we also tested the scenarios where the majority of the participants were malicious, trying to vandalize the system performance by injecting false data at varied rates.

#### Mean Absolute Error (MAE)

The Mean Absolute Error is calculated as follows.

(20) $$MAE=abs(V_{i}-\hat{V_{i}})$$

where $V_{i}$ is the original value and $\hat{V_{i}}$ denotes the predicted value by a method.

**Figure 6:**
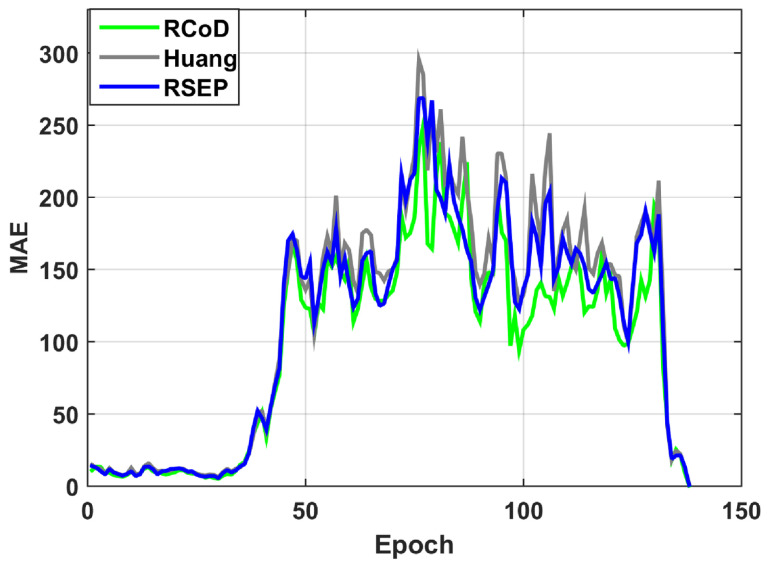
Mean Absolute Error trend in the presence of on–off attack.

**Figure 7:**
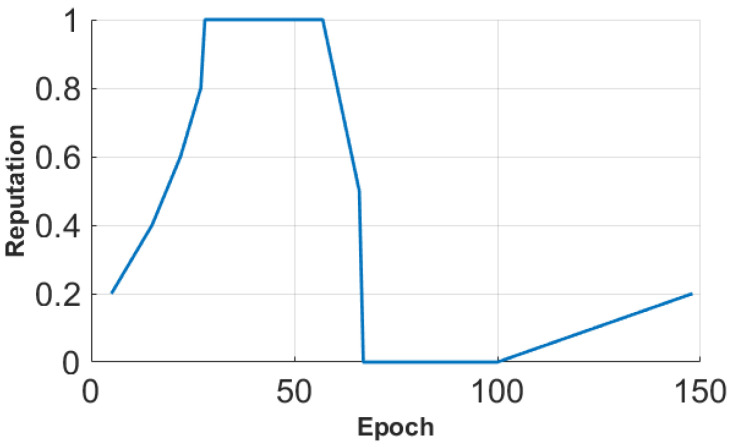
Change in reputation for an on–off attacker.

**Figure 8:**
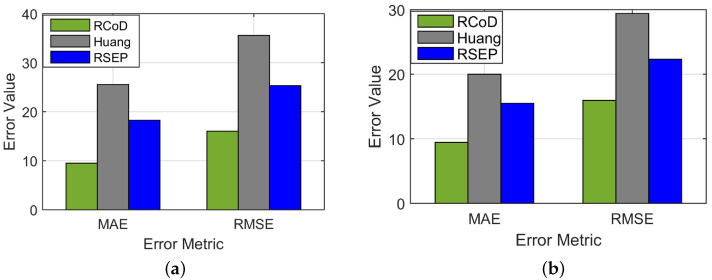
Mean Absolute Error and RMSE in the presence of (**a**) On–off attack and (**b**) 55 Data-corruption attackers.

In Figure 6, we show the change in Mean Absolute Error (MAE) change per epoch in the presence of an on–off attack. Our method RCoD incurred less MAE throughout time. When the malicious participants inject false data after achieving the highest reputation (=1.0), the MAE becomes immense for RSEP [32] and Huang [19]. The reason behind this is that the state-of-the-art methods were not able to detect the data imprecision contributed by the malicious participant who continuously contributed correct data in the past, thus assuming s/he is an honest participant. The false data injection began from epoch 80. Though at the beginning of the on–off attack, our method incurred a high MAE score, causing a sudden spike in Figure 6, RCoD was able to detect the malicious behavior and remove the participant from the trusted participant list. In Figure 7, the change in reputation for an on–off attacker is depicted. It can be observed that the reputation value dropped steeply after attaining the peak value (=1.0). Also, the growth of the reputation is slower than the decay rate. Even after behaving well after around epoch 100, the reputation value did not increase much to be included in the trusted participant list. Moreover, Figure 8a depicts the average MAE value incurred by the methods throughout the seven days. Throughout the seven-day experiment, our RCoD method outperformed RSEP and Huang. Our method achieved 47.98% less MAE than RSEP in the presence of the on–off attack. Also, RCoD incurred 62.82% less MAE than Huang.

**Figure 9:**
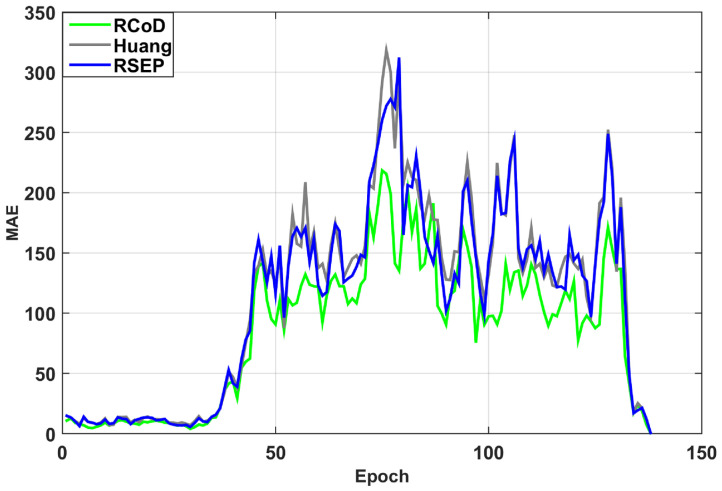
MAE Trend for 85 Malicious Nodes.

**Figure 10:**
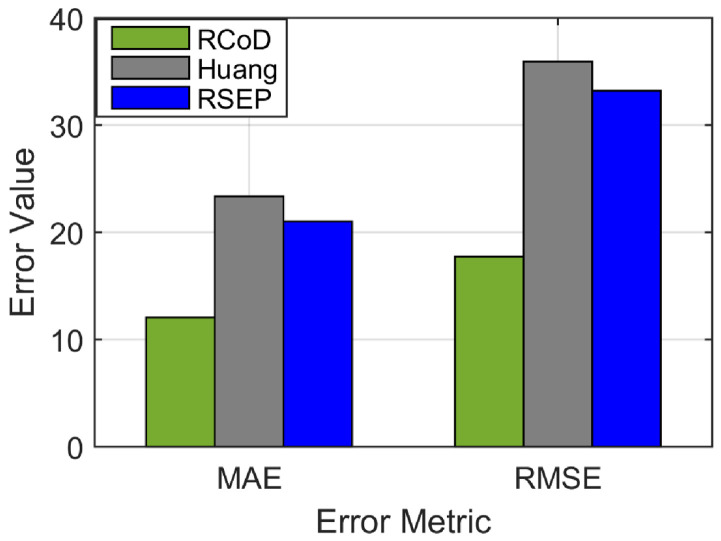
Average MAE and RMSE where the malicious node is the majority.

We also want to investigate the data-corruption attack. RCoD achieved, on average, 55.45% less MAE than Huang and 48.82% less than RSEP in the presence of varied data-corruption attackers. In Figure 9, the prediction performance of our method RCoD, Huang, and RSEP are shown. In this case, the majority of the participants who reported data were malicious and injected false data at a varied error rate ranging from 25 to 75%. This is the worst-case scenario as the number of malicious participants (60%) exceeds the number of honest participants. The state-of-the-art methods fail to predict data accurately in this scenario such as our data prediction performance. The reason behind this is that in these works, the majority of malicious participants were able to manipulate the fused data in this scenario. On average, from Figure 10, it can be seen that our method incurred 42.61% and 48.33% improvement in terms of less MAE over the RSEP and Huang, respectively.

#### Accuracy, Precision, Recall and F1 Score

We measure the performance of our data-fusion mechanism quantitatively by calculating the accuracy, precision, recall, and F1 Score. Accuracy is the ratio of correct identification of honest or malicious participants among all the detections. A higher value of accuracy insinuates the effectiveness of the method.

(21) $$Accuracy=\frac{TP+TN}{TP+FP+TN+FN}\;$$

**Figure 11:**
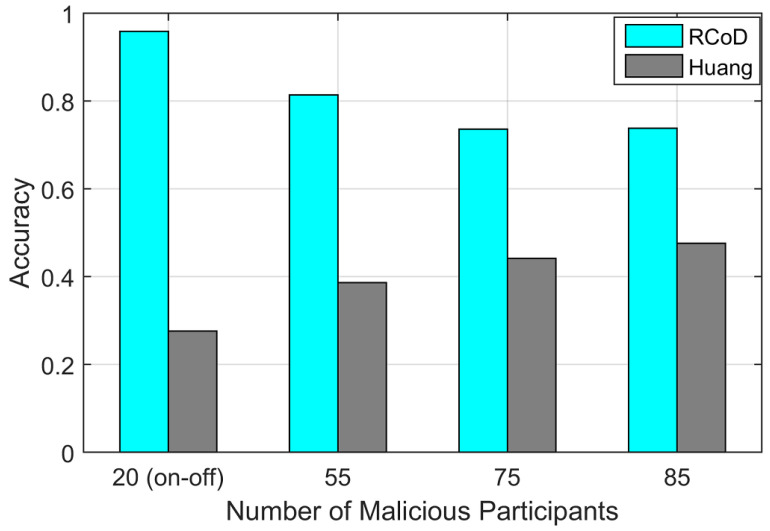
Accuracy vs malicious node.

Here, TP denotes the number of participants correctly identified as honest, and FP denotes the number of participants identified as honest but originally malicious. TN denotes the number of participants identified correctly as malicious, and FN is the number of honest participants detected as malicious. Our method was able to receive 74% accuracy in the worst-case scenario (60% malicious). The state-of-the-art methods were not able to predict data accuracy like RCoD since, for them, the majority of malicious participants were able to manipulate the overall data in this scenario. Figure 11 shows that our method achieved high accuracy. In comparison to Huang [19], our RCoD method achieved 49.82% better accuracy on average. The average accuracy value achieved by RCoD is 80%. Table 3 displays the confusion matrix incurred by RCoD for data corruption attack.

(22) $$Precision=\frac{TP}{TP+FP}\;$$

(23) $$Recall=\frac{TP}{TP+FN}\;$$

**Figure 12:**
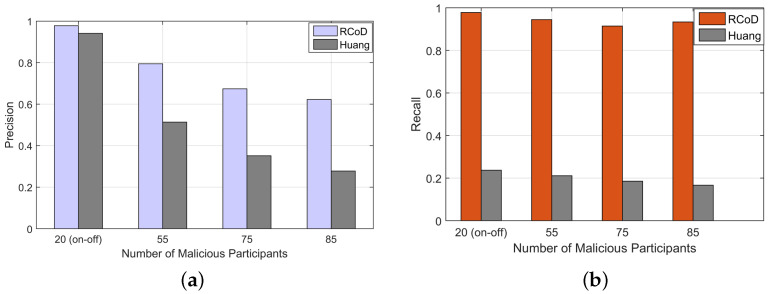
(**a**) Precision vs number of malicious node and (**b**) Recall vs malicious participants.

Figure 12a shows the achieved precision values calculated using Equation (22) [72]. On average, our method achieved a precision value of 0.77, which is 35.58% higher than Huang. Moreover, in Figure 12b, we can see the recall value in the presence of a different number of malicious participants. In the presence of 85 malicious participants, RCoD achieved a recall value of 0.93. The average recall value achieved by our method is 0.94. It represents that our algorithm successfully identified the honest participants with, on average, 94% cases. Our method outperformed Huang in terms of recall by 78.8%.

**Figure 13:**
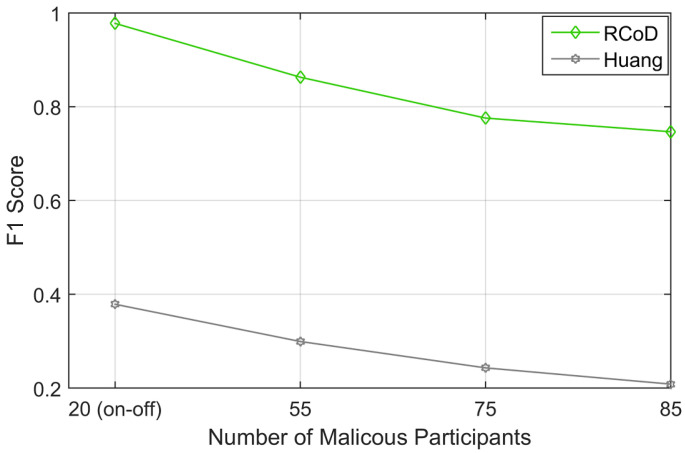
F1 Score vs malicious node.

Figure 13 shows the F1 Score achieved by RCoD and Huang in the presence of a different number of malicious participants. F1 Score is calculated using Equation (24). In the case of an on–off attack, RCoD successfully detected the data anomaly, thus achieving an F1 Score of 0.98. Furthermore, our method was able to detect the malicious participants with a decent F1 score (=0.75) in the scenario where malicious participants supersede the number of honest participants. In this scenario, the F1 Score incurred by Huang was only 0.21. Our method achieved at least 61.27% better F1 Score than Huang. The average F1 Score encountered throughout the experiments was 0.84, which indicates the high classification accuracy of our RCoD method.

(24) $$F1=2\times \frac{Precision\times Recall}{Precision+Recall}\;$$

#### AUC and Specificity

We calculated AUC and Specificity to measure the efficacy of the malicious node-detection performance of our method. AUC refers to the avoidance of false classification of the classifier. On the contrary, Specificity refers to the effectiveness of the method in terms of correct identification of malicious participants [72].

(25) $$AUC=0.5\times (\frac{TP}{TP+FN}+\frac{TN}{TN+FP})$$

(26) $$Specificity=\frac{TN}{TN+FP}$$

**Figure 14:**
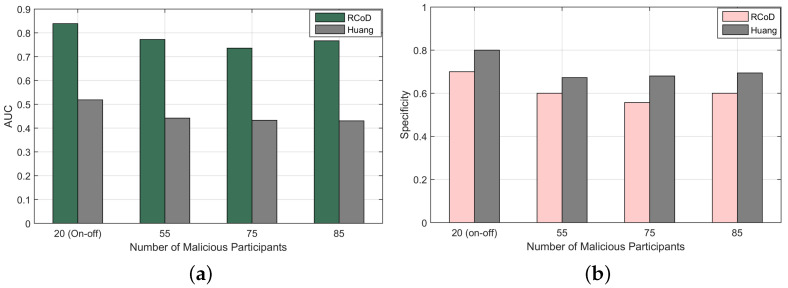
(**a**) AUC vs number of malicious node and (**b**) Specificity vs malicious participants.

Figure 14a shows the AUC value achieved in the presence of a different number of malicious participants. AUC is calculated using Equation (25). RCoD outperformed Huang in terms of AUC by 41.5% on average. It can be observed that in the presence of 60% malicious participants, the AUC value is 0.76. It insinuates that our algorithm was successfully able to avoid incorrect classification of participants even where the majority of the participants are malicious. For the on–off attack, the achieved AUC value is 0.84. Furthermore, Figure 14b depicts the achieved specificity values. Even though our main goal is the proper identification of honest participants to ensure the data accuracy of the overall system, the algorithm could identify the malicious participants accurately with an average Specificity value of 0.61. In the presence of an on–off attacker, our method could properly identify the malicious participants in 70% of cases.

#### Breaking Point of RCoD

We experimented in the presence of **88 malicious participants among the total of 145 participants** to show the breaking point of our method. Here, the number of malicious participants is over 60% of the total participants. Table 4 displays different performance metrics (e.g., Precision, Recall, Accuracy, F1 Score, AUC, and Specificity) in the presence of 88 malicious participants. The performance degrades a lot in comparison to the presence of less than 60% malicious participants. Our Reputation-based context-aware data-fusion (RCoD) method fails to identify properly the honest participants when the number of malicious participants is 88. From Table 3, we can see that our method was able to identify 19 honest participants accurately (TP) among the 57 original honest participants. This insinuates that our method failed to classify honest and malicious participants properly when the number of malicious participants exceeds 60% of the total. With a 1% increase over 60% malicious participants, the performance degrades significantly. Thus, our RCoD is resilient against up to 60% of malicious participants.

#### Root Mean Square Error (RMSE)

**Figure 15:**
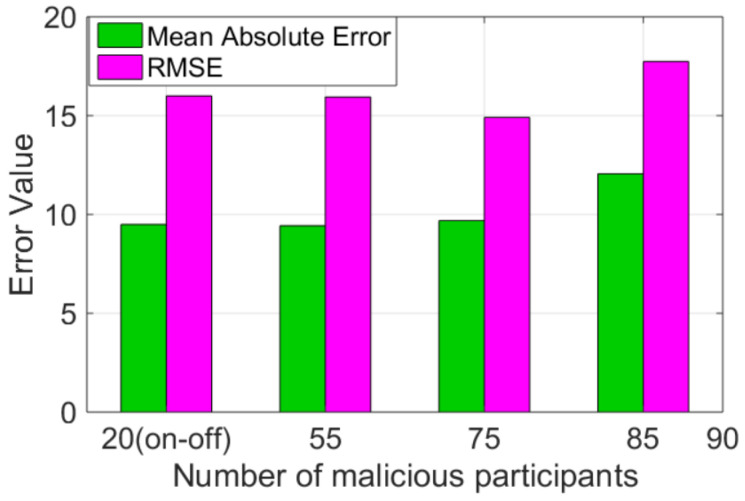
Average MAE and RMSE incurred by RCoD.

We calculated Root Mean Square Error (RMSE) as a prediction metric. It insinuates the prediction error of a method. RMSE is a standard metric to evaluate the accuracy of the prediction model [60].

(27) $$RMSE=\sqrt{\frac{1}{n}\sum_{i=1}^{n}{\left({\hat{V}}_{i}-V_{i}\right)}^{2}}\;$$

where ${\hat{V}}_{i}$ is the predicted value, $V_{i}$ is the original value and *n* is the number of epochs. In Figure 8, we show the RMSE incurred by our RCoD and RMSE method in the case of an on–off attack. Figure 8 and Figure 10 depict the RMSE value incurred by the methods (RCoD, Huang, and RSEP) where 55 and 85 malicious attackers among the total 145 participants performing data-corruption attack. Our method outperformed Huang and RSEP by 45.81% and 28.6%, respectively, in the presence of 55 malicious participants. On the other hand, in the worst-case scenario where the majority of the participants are malicious, the performance of RCoD is noteworthy. Our method outperformed the closest competitor, RSEP, by 46.58%. Furthermore, our method incurred 50.62% less RMSE than Huang. We make similar observations for RCoD under a varied number of malicious participants infiltrating the system, as shown in Figure 15. The average prediction error incurred by our method is reasonably low.

**Figure 16:**
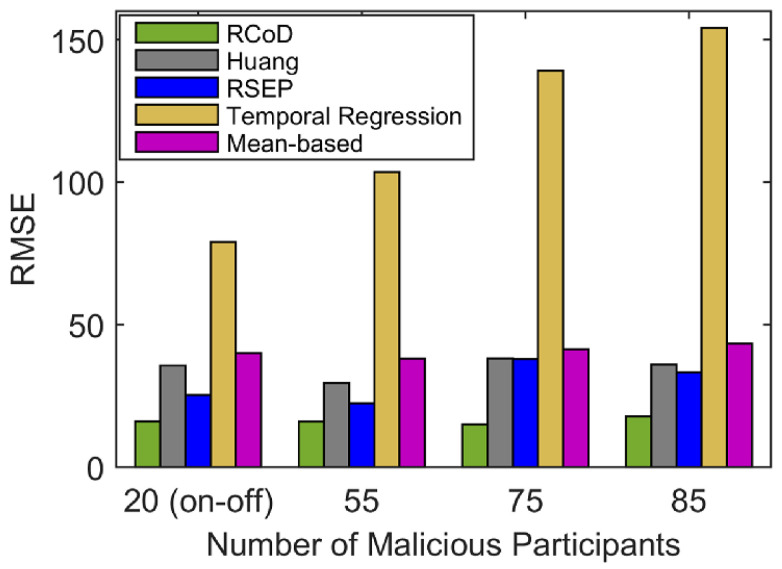
Average RMSE Comparison.

Next, Figure 16 presents the RMSE value incurred by our RCoD, Huang [19], RSEP [32], mean-based [56,57], and temporal regression [58] for an on–off attack and data-corruption attack. We tested against a different number of malicious participants. Our method outperformed the closest competitor, RSEP, by incurring 43.15% less RMSE on average. In the presence of varied malicious participants, RCOD incurred at least 45.8% and at most 60.88% less RMSE than Huang. The performance superiority over temporal regression and mean-based is noteworthy. The reason behind the poor performance of other methods can be explained as they do not incorporate feedback from reputable participants to validate the reported data. Also, our dynamic trusted set and the distribution of contribution score highly reflect the ground truth data. Furthermore, the Contextual Hidden Markov Model exploits the contextual relationship among the participants in the data prediction method. Hence, our RCoD method achieves better prediction than the compared state-of-the-art methods in the presence of a high number of malicious participants.

## 7. Conclusions and Future Work

In this article, we developed an online method for data quality prediction in mobile IoT that considers spatial, temporal, and context relationships among participants. We implemented our methods on the Beijing air-quality dataset. Most state-of-the-art methods assume the presence of malicious participants ranging from 20% to 50%. However, we also tested scenarios where the majority of participants were malicious, trying to vandalize the system performance by injecting false data at varied rates. Our RCoD could tolerate the presence of up to 60% malicious participants located in a close vicinity. We tested in the presence of different high numbers (55, 65, 75, and 85 out of 145) of participants injecting false data. In the case of an on–off attack, RCoD successfully detected the data anomaly by achieving an F1 Score of 0.98. Our method exhibited its resilience by achieving 74% accuracy in the worst-case scenario (60% malicious participants). In this case, our RCoD outperformed the closest competitors, RSEP and Huang, by incurring, on average, 43.15% and 53.08% less RMSE, respectively. The state-of-the-art methods were not able to achieve prediction data accuracy like our RCoD since, for them, the majority of malicious participants were able to manipulate the overall data in this scenario. The success of our approach lies in the integration of dynamic trust evaluation of the sensed data, which allows us to defend against data-corruption and on–off attacks, as well as identify malicious or honest participants based on their reported data in real time.

Our method makes data-injection attacks harder, but it is breakable by collusion among malicious participants. In the future, we will extend this work considering collusion attacks and trial-and-error attacks of malicious participants. Future research should include the detection of emerging threats and designing a data-augmentation algorithm.

## Figures and Tables

**Table 1:** Notations and Their Description.

Notation	Description
pc	Participant count
$C_{i}^{k}$	Sensor data of type k contributed by participant i
$\alpha_{i}^{j}$	Contribution score of participant i with sensor type j
trustedSet	Set of trusted participants
initEpoch_end	Ending epoch for initial trusted set calculation
$\beta_{i}$	Proximity score of participant i
$Rep(P_{f})$	Reputation of feedback provider
$Rep(P_{i})$	Reputation of participant i
$Num_{FP}$	Number of feedback providers
$Feed_{f}\left(P_{i}\right)$	Feedback from participant f regarding data reported by participant i
$\theta_{i}$	Feedback score of participant i
$\delta_{i}^{j}$	Willingness of participant i for sensor data type j
$Context_{trustedSet}$	Context value of the trusted set of participants
$\gamma_{i}^{j}$	Context score of participant i with sensor type j
$t_{duration}$	Application-dependent threshold
$\lambda_{i}$	Timeliness score of participant i
$Trust(C_{i}^{j})$	Trust of participant i with sensor type j
TPA	Number of Trusted Participants
reputation_table	Global 3-column table for storing participant reputation score
*C*	Context matrix
*N*	Number of participants
*Y*	Reported data matrix
*T*	Final epoch
OV	Observation dependence vector
CV	Hidden contextual dependence vector

**Table 2:** Parameter Setting.

Parameter	Value	Parameter	Value
ine $w_{1}$	0.4	$w_{5}$	0.15
$w_{2}$	0.15	$w_{6}$	0
$w_{3}$	0	$w_{7}$	0.3
$w_{4}$	0	threshold	0.5
Epoch Length	1 h	initEpoch_end	24 h
reward_val	0.2	punish_val	0.5

**Table 3:** Confusion Matrix for Data-Corruption Attack.

Number of Malicious Participants	TP	TN	FP	FN
55	85	33	22	5
75	64	39	31	6
85	56	51	34	4
88	19	53	35	38

**Table 4:** Performance Metric in the presence of 88 (greater than 60%) malicious participants.

Precision	Recall	Accuracy	F1 Score	AUC	Specificity
0.3518	0.3333	0.4965	0.3423	0.4678	0.6022

## Data Availability

The data that support the findings of this study are openly available at https://www.microsoft.com/en-us/research/publication/u-air-when-urban-air-quality-inference-meets-big-data/ (accessed on 15 October 2024).
